# A39 HEALTHY FIRST-DEGREE RELATIVES FROM MULTIPLEX FAMILIES VERSUS SIMPLEX HARBOR A HIGHER RISK OF DEVELOPING CROHN'S DISEASE AND ARE ASSOCIATED WITH SUBCLINICAL INFLAMMATION AND ALTERED MICROBIOME COMPOSITION

**DOI:** 10.1093/jcag/gwac036.039

**Published:** 2023-03-07

**Authors:** P Olivera, H Martinez-Lozano, H Leibovitzh, M Xue, W Xu, O Espin-Garcia, K Madsen, J Meddings, D Guttman, A Griffiths, H Huynh, D Turner, R Panancionne, H Steinhart, G Aumais, K Jacobson, D Mack, J Marshall, P Moayyedi, S -H Lee, W Turpin, K Croitoru

**Affiliations:** 1 Division of Gastroenterology & Hepatology, Zane Cohen Centre for Digestive Diseases, Mount Sinai Hospital; 2 Temerty Faculty of Medicine; 3 Division of Biostatistics, Dalla Lana School of Public Health, University of Toronto, Toronto; 4 University of Alberta, Edmonton; 5 Department of Medicine, Cumming School of Medicine, Calgary; 6 Department of Cell & Systems Biology; 7 Centre for the Analysis of Genome Evolution & Function; 8 IBD Center, The Hospital for Sick Children, Department of Paediatrics, University of Toronto, Toronto; 9 Division of Gastroenterology and Nutrition, Department of Pediatrics, Faculty of Medicine and Dentistry, University of Alberta, Edmonton, Canada; 10 The Juliet Keidan Institute of Pediatric Gastroenterology and Nutrition, Shaare Zedek Medical Center, The Hebrew University of Jerusalem, Jerusalem, Israel; 11 Inflammatory Bowel Disease Clinic, Division of Gastroenterology and Hepatology of Gastroenterology, University of Calgary, Calgary; 12 Department of Medicine, Hôpital Maisonneuve-Rosemont, Montreal University, Montreal; 13 British Columbia Children's Hospital, British Columbia Children's Hospital Research Institute, University of British Columbia, Vancouver; 14 Division of Gastroenterology, Hepatology & Nutrition, Children's Hospital of Eastern Ontario, University of Ottawa, Ottawa; 15 Department of Medicine, McMaster University, Hamilton, Canada

## Abstract

**Background:**

**Healthy individuals within families with multiple affected members (multiplex families) with Crohn’s disease (CD) have a notably high risk of developing CD. No large prospective pre-disease cohort has assessed differences in preclinical intestinal inflammation, permeability, fecal microbiome, and genetics in healthy at-risk subjects from multiplex families.**

**Purpose:**

We aimed to assess differences in subclinical gut inflammation, genetic risk, gut barrier function, and fecal microbiota composition between first-degree relatives (FDRs) from families with 2 or more affected members (multiplex) and families with only one affected member (simplex). Also, we aimed to assess the risk of future CD onset in subjects from multiplex versus simplex families.

**Method:**

We utilized the GEM Project cohort of healthy FDRs of CD patients. Subclinical gut inflammation was assessed using fecal calprotectin (FCP) at recruitment. Gut barrier function was assessed using the lactulose-to-mannitol ratio (LMR). For assessment of the CD-related genetic risk, CD-polygenic risk scores (CD-PRS) were calculated. Microbiome composition was assessed by sequencing fecal 16S ribosomal RNA. Generalized estimating equations logistic regression and LEfSe (PMID: 21702898) were used to assess the associations between multiplex status and different outcomes. A Cox proportional hazards model was used to assess time-related risk of future onset of CD.

**Result(s):**

4385 subjects were included. Median age was 17 [IQR 12-24] years, 52.9% were female, 69.4% were siblings and 30.6% were offspring. 4052 (92.4%) and 333 (7.6 %) were simplex and multiplex subjects, respectively. After adjusting for age, sex, family size, and relation to proband, multiplex status was significantly associated with higher baseline FCP (p=0.038), but was not associated with either baseline LMR or CD-PRS (p=0.19 and p=0.33, respectively).

We found no significant differences in alpha diversity (Shannon index) (p=0.57) between simplex and multiplex subjects. Beta diversity analysis assessed by the Bray-Curtis dissimilarity index did not reveal significant differences (R2=3e-04, p=0.607). The genera *Eisenbergiella*, *Eggerthellaceae uncultured*, and *Morganella*, were significantly more abundant in multiplex subjects, whereas *Lachnospira*, *Sutterella*, *Lachnospiraceae_NK4A136_group*, and *Lachnospiraceae_UCG_004* less abundant.

The risk of CD onset was significantly higher in multiplex subjects. In multivariable analysis, multiplex status at recruitment was associated with increased risk of CD onset (adjusted HR 3.41, 95% CI 1.70-6.87, p=0.00055), after adjusting for demographics, FCP, LMR, and CD-PRS.

**Conclusion(s):**

Multiplex status compared to simplex is associated with a 3.4-fold increased risk of CD onset, a higher FCP, and fecal bacterial composition. A comprehensive assessment of environmental factors that increase CD risk in multiplex families remains to be elucidated in future studies.

**Disclosure of Interest:**

None Declared

